# How Do Spike Collisions Affect Spike Sorting Performance?

**DOI:** 10.1523/ENEURO.0105-22.2022

**Published:** 2022-09-30

**Authors:** Samuel Garcia, Alessio P. Buccino, Pierre Yger

**Affiliations:** 1 Centre de Recherche en Neuroscience de Lyon, Centre National de la Recherche Scientifique, Lyon 69500, France; 2 Department of Biosystems Science and Engineering, ETH Zurich 8092, Switzerland; 3 Institut de la Vision, Sorbonne Université, Institut National de la Santé et de la Recherche Médicale, Paris 75012, France

**Keywords:** benchmark, overlapping spikes, spike collision, spike sorting

## Abstract

Recently, a new generation of devices have been developed to record neural activity simultaneously from hundreds of electrodes with a very high spatial density, both for *in vitro* and *in vivo* applications. While these advances enable to record from many more cells, they also challenge the already complicated process of spike sorting (i.e., extracting isolated single-neuron activity from extracellular signals). In this work, we used synthetic ground-truth recordings with controlled levels of correlations among neurons to quantitatively benchmark the performance of state-of-the-art spike sorters focusing specifically on spike collisions. Our results show that while modern template-matching-based algorithms are more accurate than density-based approaches, all methods, to some extent, failed to detect synchronous spike events of neurons with similar extracellular signals. Interestingly, the performance of the sorters is not largely affected by the spiking activity in the recordings, with respect to average firing rates and spike-train correlation levels. Since the performances of all modern spike sorting algorithms can be affected as function of the activity of the recorded neurons, scientific claims on correlations and synchrony should be carefully assessed based on the analysis provided in this paper.

## Significance Statement

High-density extracellular recordings allow experimentalists to get access to the spiking activity of large neuronal population, via the procedure of spike sorting. It is widely known that spike sorters are affected by spike collisions, i.e., the occurrence of spatiotemporally overlapping events, but a quantitative benchmark is still lacking. In this contribution, we perform systematic comparisons on the performance of many different spike sorters against spike collisions, showing that modern spike sorters, to different degrees, are still affected by synchronous events. Our results suggest that scientific claims on neuron correlations and synchrony should be carefully assessed as they could result from spike sorting errors.

## Introduction

Accessing the activity of large ensemble of neurons is a crucial challenge in neuroscience. In recent years, multielectrode arrays (MEAs) and large silicon probes have been developed to record simultaneously from hundreds of electrodes packed with a high spatial density, both *in vivo* (Jun et al., 2017; Angotzi et al., 2019) and *in vitro* (Berdondini et al., 2009; Frey et al., 2009). With these devices, each electrode records the extracellular field in its vicinity and can detect the action potentials (or spikes) emitted by the neighboring neurons in the tissue. In contrast to intracellular recording, extracellular recordings do not give a direct and unambiguous access to single neuron activity and one needs to further process the recorded signals to extract the spikes emitted by the different cells around the electrodes. This complex problem of source separation is termed “spike sorting.” While various solutions for small number of channels (tens at max) can be found in the large literature on spike sorting algorithms (Quiroga et al., 2004), these new devices with thousands of channels challenge the classical approach to perform spike sorting.

Recently, a new generation of spike sorting algorithms have been developed to be able to deal with hundreds (or even thousands) of channels recorded simultaneously (for recent review, see Lefebvre et al., 2016; Hennig et al., 2019). The extent to which these modern spike sorting algorithms recover all the spikes from a neuronal population is still under investigations, and might differ depending on the species, tissue, cell types, activity level. While most of the real ground truth recordings (Neto et al., 2016; Yger et al., 2018) are assessing the performance at the single cell level, to obtain an exhaustive assessment of the spike sorting performance at the population level, one must turn to use fully artificial or hybrid dataset (Buccino and Einevoll, 2020; Magland et al., 2020) to properly compare and quantify the performances of the algorithms. But even with such dataset, in most of the studies, errors are only measured as false positive (FP)/false negative (FN) rates, and the reasons behind failures of the algorithms are often overlooked.

In this study, we focused on a key property of the spike trains, at the core of most of these failures, i.e., their fine temporal correlations. Indeed, temporal correlations are ubiquitous in the brain, and the higher the number of recorded cells because of the increased density of the probes, the more prominent they are. Correlations might have an important role in population coding (for review, see Averbeck et al., 2006), but correlated activity for nearby cells results, in the extracellular signals, in overlapping activities and thus are harder to identify than isolated spikes. While pioneering work (Pillow et al., 2013) claimed that template-matching-based algorithms were more suited to recover overlapping spikes (either in space and/or time), the extent to which they are is not properly defined. In this work, our aim is to estimate how good (or bad) modern spike sorters are in recovering colliding spikes. What are the limits of the sorters, and what are the key parameters of the recordings and/or of the neurons that could influence these numbers?

## Materials and Methods

All the code used to generate the figures is available at https://spikeinterface.github.io/.

### Simulated datasets

We used the MEArec simulator (Buccino and Einevoll, 2020) to generate 30-min-long synthetic ground truth recordings. In brief, MEArec uses biophysically detailed multicompartment models to simulate the extracellular action potentials, or so called “templates.” For this study, we used 13 cell models from layer 5 of a juvenile rat somatosensory cortex (Markram et al., 2015; Ramaswamy et al., 2015) to get a dictionary of biologically plausible templates. Given this database, we took the layout of a NeuroNexus probe (A1x32-Poly3-5 mm-25s-177-CM32 with 32 electrodes in three columns and hexagonal arrangement, a x-pitch and y-pitch of 18 and 22 μm, respectively, and an electrode radius of 7.5 μm), and randomly positioned 20 cells in the vicinity of the probe, so that every simulated neuron has a unique template (i.e., average extracellular action potential). Templates are then combined with spike trains and slightly modulated in amplitude to add physiological variability. Additive uncorrelated Gaussian noise is finally added to the traces. We generated simulated recordings with 20 neurons randomly positioned in front of the probe, a noise level of 5 μV and a sampling rate of 32 kHz. To obtain more robust results, we generated five recording per conditions with various random seeds. The spike times were kept unchanged, but the positions and the templates of the 20 neurons were changed in each of the individual recording. This allowed us to populate the distribution of cosine similarities between pairs.

### Generating spike trains with controlled correlations

To generate the recordings with various firing rates and correlations levels, we used the mixture process method described in (Brette, 2009). Since by default the method generates controlled cross-correlograms with a decaying exponential profile, we modified it to generate cross-correlograms with a Gaussian profile, to have more synchronous firing for small lags. By setting three different rate levels (5, 10, and 15 Hz) and three different correlation levels (0%, 10%, and 20%) this gave rise to nine conditions, so to 45 recordings in total (five recordings per conditions; see above).

### Template similarity

We define the template for neuron *i* as $T_{i}\in {\mathbb{R}}^{TxC}$, with *T* representing the number of samples and *C* the number of channels. After flattening the template by concatenating the signals from different channels ( $T_{i}^{f}\in {\mathbb{R}}^{T\cdot C}$), the similarity between two neurons *i* and *j* is quantified via the cosine similarity defined as follows:

(1) $$similarity=\frac{T_{i}^{f}\cdot T_{j}^{f}}{\left||T_{i}^{f}||||T_{j}^{f}|\right|}=cos(\theta ),$$

where *θ* is the angle between the two (*T* · *C*)-dimensional vectors $T_{i}^{f}$ and $T_{j}^{f}$. The cosine similarity is therefore bounded between −1 (templates are anti-parallel) and 1 (templates are parallel). A cosine similarity of 0 means that the templates are orthogonal.

### Spike sorters

All the spike sorters used in this study were run using the SpikeInterface framework (Buccino et al., 2020), with default parameters. The following are the exact versions that we used for the different spike sorters: Tridesclous (1.6.4), Spyking-circus (1.0.9; Yger et al., 2018), HerdingSpikes (0.3.7; Hilgen et al., 2017), Kilosort (v1, 2, or 3; Pachitariu et al., 2016), YASS (2.0; Lee et al., 2020), IronClust (5.9.8; Chung et al., 2017), and HDSort (1.0.3; Diggelmann et al., 2018). The desktop machine used has 36 Intel Xeon(R) Gold 5220 CPU @ 2.20 GHz, 200Go of RAM and a Quadro RTX 5000 with 16 Gb of RAM as a GPU.

### Spike sorting comparison

All the quantitative metrics between the results of the spike sorting software and the ground-truth recording were made via the SpikeInterface toolbox.

When comparing a spike sorting output to the ground-truth spiking activity, first an agreement score between each pair of ground-truth and sorted spike trains is computed as:

$$score_{ij}=\frac{\#n_{matches}}{\#n_{i_{gt}}+\#n_{j_{sorted}}-\#n_{matches}},$$

where $\#n_{i_{gt}}$ and $\#n_{j_{sorted}}$ are the numbers of spikes in the *i*-th ground-truth spike train and the *j*-th sorted spike trains, respectively. #*n_matches_* is the number of spikes within 0.4 ms between the two spike trains.

Once scores for all pairs are computed, a Hungarian assignment is used to match ground-truth units to sorted units (Buccino et al., 2020). All spikes from matched spike trains are then labeled as: true positive (TP), if the spike is found both in the ground-truth and the sorted spike train; FP, if the spike is found in the sorted spike train, but not in the ground-truth one; and FN, if the spike is only found in the ground-truth spike train.

After labeling all matched spikes, we can now define these unit-wise performance metrics for each ground-truth unit that has been matched to a sorted unit:

(2) $$accuracy=\frac{\#TP}{\#TP+\#FP+\#FN}$$

(3) $$precision=\frac{\#TP}{\#TP+\#FP}$$

(4) $$recall=\frac{\#TP}{\#TP+\#FN}.$$

The global accuracy, precision, and recall values shown in Figure 2*D* are the average values of the performance metrics computed by unit.

Using the unit metrics and the output of the matching procedure, we can further classify each sorted unit as:

Well detected: sorted units with an accuracy ≥0.8;

False Positive: sorted units that are not matched to any ground-truth unit and have a score <0.2;

Redundant: sorted units that are not the best match to a ground-truth unit but have a score ≥0.2;

Overmerged: sorted units with a score ≥0.2 with more than one ground-truth unit.

In order to generate the spike lag versus recall figures (e.g., Figs. 3-6) we expanded the SpikeInterface software with several novel comparison methods and visualization widgets. In particular, we extended the ground-truth comparison class to the CollisionGTComparison, which computes performance metrics by spike lag. In addition to the agreement score computation and the matching described in the previous paragraphs, this method first detects and flags all “synchronous spike events” in the ground-truth spike trains. Two spikes from two separate units are considered to be a “synchronous spike event” if their spike times occur within a time lag of 2 ms. The synchronous events are then binned in 11 bins spanning the [–2, 2] ms interval, and the collision recall is computed for each bin. With a similar principle, we implemented the CorrelogramGTComparison to compute the lag-wise relative errors in cross-correlograms between ground-truth units and spike sorted units.

## Results

### Generation of the ground-truth recordings

**Figure 1.**
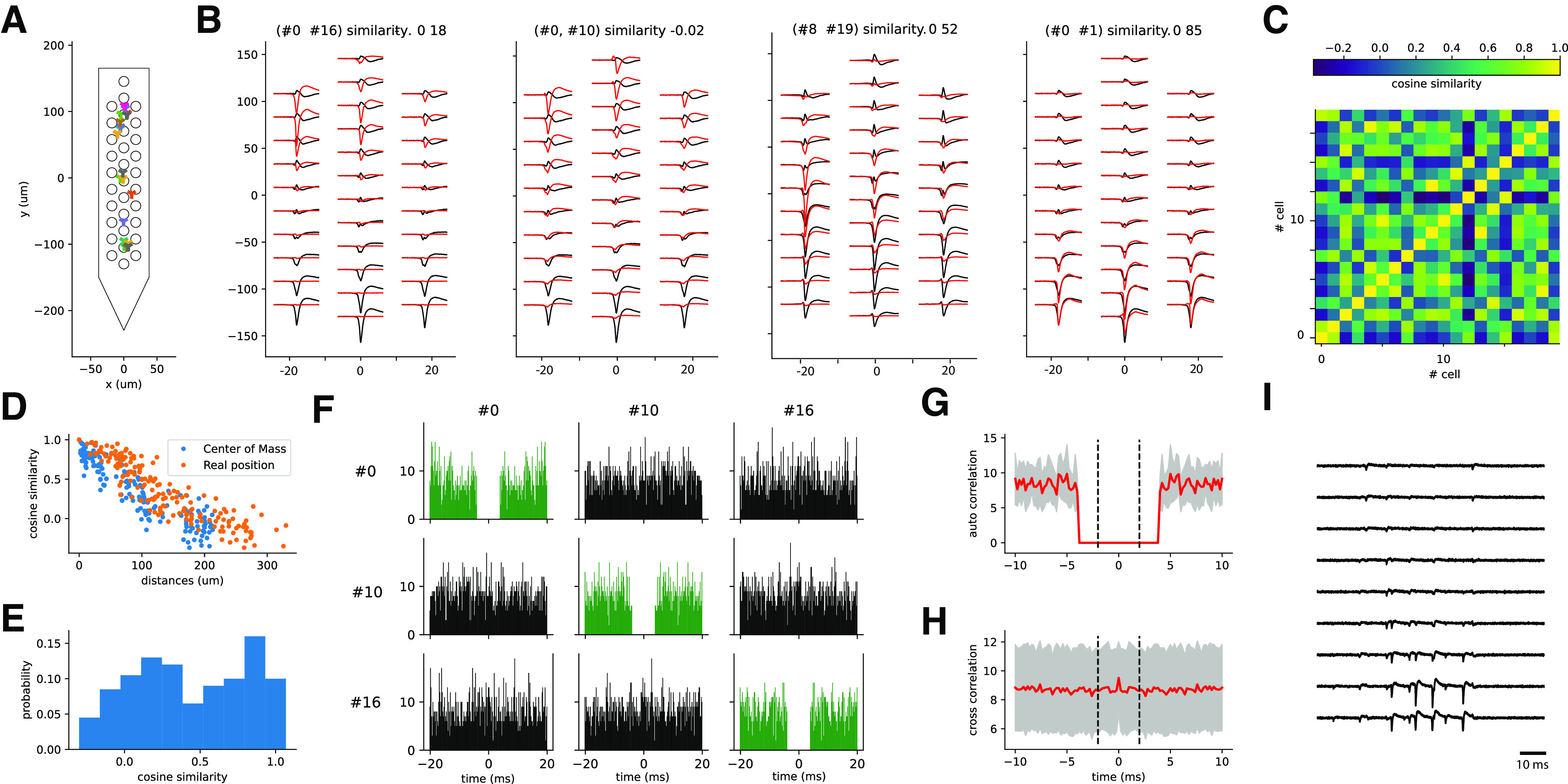
Generation of the synthetic recordings. ***A***, A total of 20 cells are randomly placed in front of a 32-channel NeuroNexus probe layout. The plot shows the location of each cell for one recording. ***B***, Sample template pairs generated by neurons with different cosine similarity values. ***C***, Cosine similarity matrix between all pairs of templates for a sample recording. ***D***, Cosine similarity as function of the distance between the neurons, either using the real position from the simulations (orange circles), or the estimated barycenter of the templates (blue circles). ***E***, Histogram of the cosine similarity distribution from one of the simulated recordings. ***F***, Cross-correlograms and auto-correlograms for three sample spike trains. ***G***, Average auto-correlograms of all units (red line, gray area represents the SD). ***H***, Average cross-correlogram over all pairs of neurons (red line, gray area represents the SD around the mean). ***I***, Sample traces from 10 channels of one synthetic recording.

To test how robust the recently developed spike sorting pipelines are against spike collisions (Pachitariu et al., 2016; Chung et al., 2017; Hilgen et al., 2017; Yger et al., 2018; Lee et al., 2020), we generated synthetic datasets using the MEArec simulator (Buccino and Einevoll, 2020; see Materials and Methods). More precisely, we took the layout of a NeuroNexus probe with 32 electrodes in three columns and hexagonal arrangement, and randomly positioned 20 cells in the vicinity of the probe (see Fig. 1*A*), so that every simulated neuron has a unique template (i.e., average extracellular action potential). Figure 1*B* shows three sample templates with, respectively, low, almost null, and high similarity. The similarity between templates is computed as the cosine similarity of the flattened signals (see Materials and Methods) and the random generation of the positions and cell types of the simulated neurons (and thus of the templates) gives rise to the similarity matrix displayed in see Figure 1*C*. This similarity, as expected, decreases with the distance between the neurons, computed either from the ground-truth positions of the cells from the simulation or estimated as the barycenters of the templates (Fig. 1*D*). The more negative the similarity is, the more templates are “in opposition”; the more positive it is, the more templates are “similar.” A similarity close to 0 means that templates do not overlap and are strongly orthogonal, i.e., dissimilar. Importantly, the simulations allowed us to cover rather uniformly the space of cosine similarities between templates, which will be used to assess the performance of spike sorters during collisions (Fig. 1*E*).

To generate the spike trains, we first used a simple approach that forced all the neurons to fire as independent Poisson sources at a fixed and homogeneous firing rate of 5 Hz. To make the simulation more biologically plausible, we pruned all spikes breaking a refractory period violation of 4 ms. The resulting auto-correlograms and cross-correlograms for three sample units are shown in Figure 1*F* (auto-correlograms are in green on the diagonal), while Figure 1*G*,*H* display the average (red line) and standard deviation (SD) (gray shaded area) auto-correlation and cross-correlation among all units, respectively. A sample snippet of the generated traces from one recording is shown in Figure 1*I*, for a subset of 10 channels out of 32. Because of the independence of the Poisson sources, both the average cross-correlograms (Fig. 1*G*) and auto-correlograms, outside the ±4 ms used as refractory period (Fig. 1*H*), are flat.

### Global performance of the spike sorters

**Figure 2.**
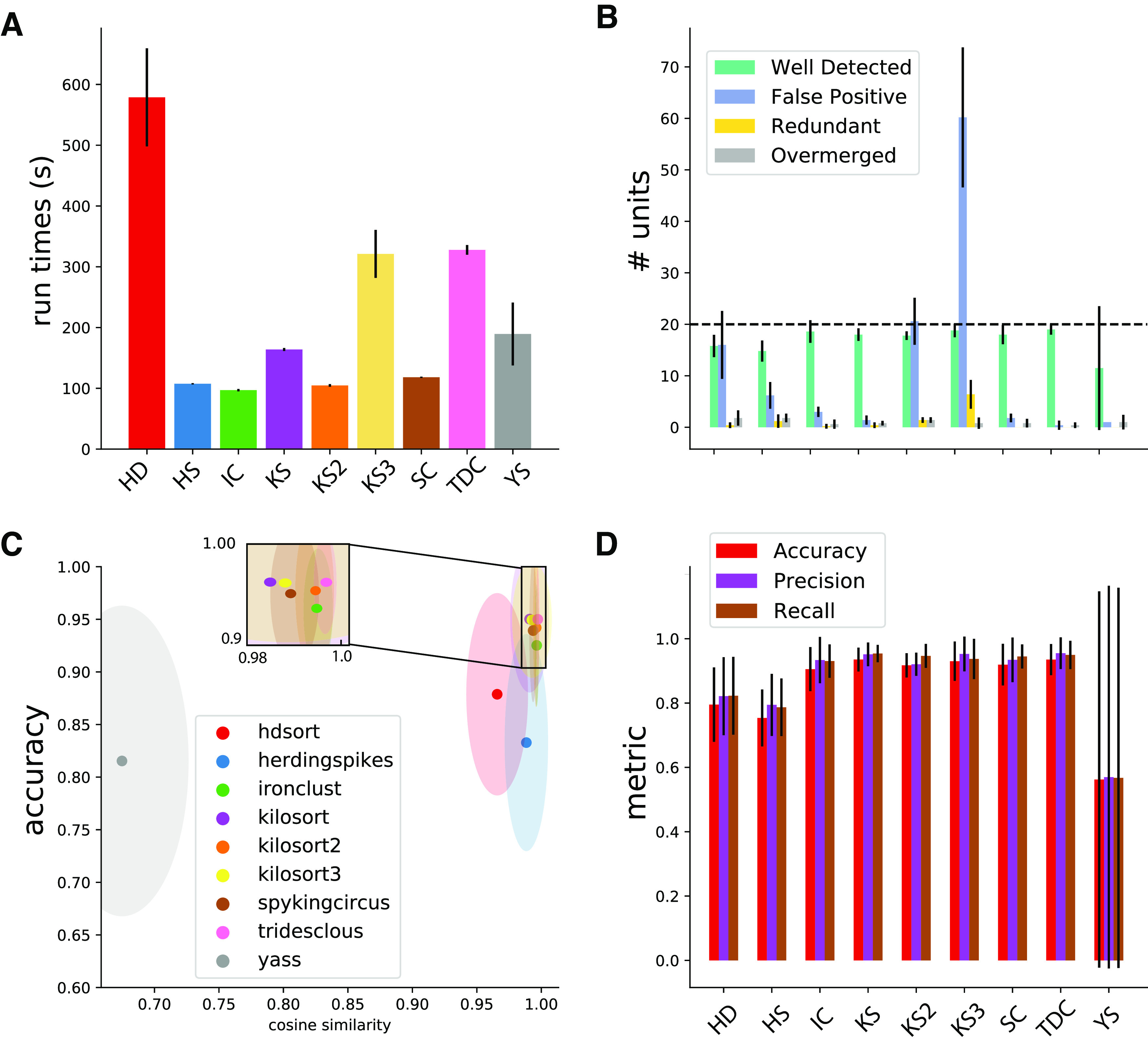
Spike sorting performance. ***A***, Average run times over five different recordings (see Materials and Methods) for all the tested sorters. Errors bars indicate the SD over multiple recordings. ***B***, Average number of cells found by the sorters that are either well detected, redundant, overmerged, or considered as FP (see Materials and Methods). Error bars indicates SD over multiple recordings. ***C***, The average cosine similarity between templates found by the sorters and ground-truth templates, as function of the accuracy for the given neurons. Ellipses shows standard error of the means in cosine similarity (*x*-axis) and accuracy (*y*-axis). ***D***, Average metrics (accuracy, precision, recall; see Materials and Methods) for all the sorters. Error bars show SD over multiple recordings.

In order to assess the global performances of the sorting procedure on our synthetic datasets, we generated five recordings with various random seeds and averaged the results. Figure 2 summarizes the main findings. First, we noticed that, as seen in Figure 2*A*, the run time was roughly constant across sorters, except for HDSort, with its higher run time. The number of well detected units is similar among sorters, as shown in Figure 2*B*, but it is worthwhile noticing that Kilosort 3 is the only sorter producing many FP and redundant units (see Materials and Methods for classification of units). Kilosort 2 and HDSort also identify more FP than well detected units. Importantly, we did not perform any curation of the spike sorting output, but we consider the raw output of each sorter as is.

To check whether all sorters correctly discovered all templates, we computed the cosine similarity between the ground-truth templates from the simulations and the ones found by the sorters, comparing such a metric with the accuracy. By doing so, we wanted to rule out the fact that the sources of the errors could primarily be because of problems in the clustering. Indeed, if the spike sorting algorithms are properly behaving, they should find templates very similar to the ground-truth ones. As it can be seen in Figure 2*C*, all sorters are on average finding the correct templates, with the notable exception of YASS (in gray) and to some less extent HDSort (in red). The average cosine similarity between found and ground-truth templates is larger than 0.97 for most template-matching-based sorters (Spyking-circus, Kilosort 1/2/3, IronClust, Tridesclous), so we can safely assume that most of the errors are not because of the clustering step. Moreover, the overall accuracy of most of the spike sorters is relatively high (∼0.95), except for HDSort and HerdingSpikes which yield lower scores (Fig. 2*D*). However, this averaged number does not tell us anything regarding the nature of these errors. While this error rate might seem low, it is likely that it is crucial, since it can potentially originate from the collisions, and thus from the correlations among neurons.

### Spike sorting performance is affected by spike collisions

**Figure 3.**
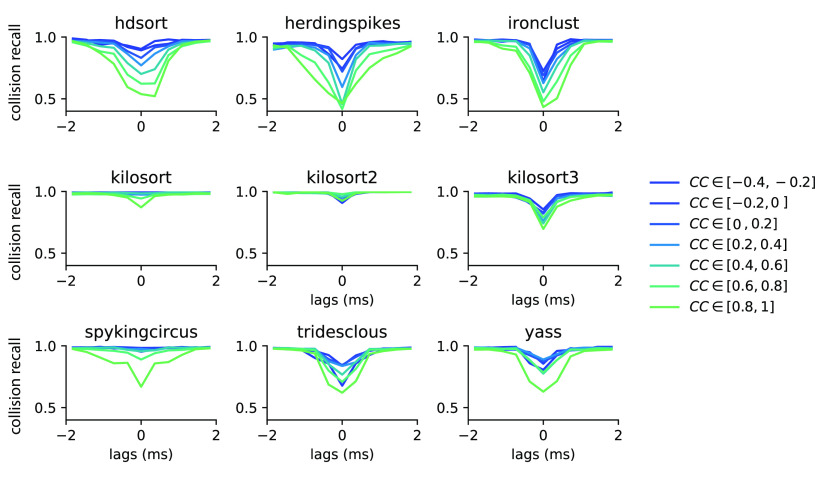
Collision recall per sorter. Error (quantified as the collision recall; see Materials and Methods) for various sorters and for all possible lags (between −2 and 2 ms), as function of the similarity between the pairs of templates (color code). All curves are averaged over multiple pairs and multiple recordings (see Materials and Methods).

Using fully synthetic recordings with exhaustive ground truth, we can look at how good individual spike sorters perform specifically with respect to spatiotemporal collisions. To do so, we computed the collision recall (see Materials and Methods) as a function of the lag between two spikes, for a given pair of neurons. By averaging over multiple pairs of ground-truth neurons with similar template similarity (and over multiple recordings; see Materials and Methods), we can obtain a picture of how accurate the sorters are specifically with respect to the spike time lags and the similarities between templates. Figure 3 displays the collision recall per sorter as a function of the lag (*x-axis*), colored by the similarity between templates. Each panel shows the performance of a different spike sorter. One can immediately see that only few sorters are able to accurately resolve lag correlations that are close to zero, even when templates are strongly orthogonal (low cosine similarity). This is the case for Kilosort 1 and 2, and for Spyking-circus, all of which use a template-matching procedure that should theoretically explain this behavior. It is worthwhile noting that the decrease in performance for Kilosort 3 is surprising, since the authors confirmed the software is using the exact same template-matching procedure than in previous versions. This means that errors are likely originating either from subtle variations in the preprocessing steps, and/or in the clustering that has been changed and thus might lead to slight differences in the templates. However, while performances are still good for Kilosort 1 and 2 even when the average cosine similarity between pairs is increased, they slightly degrade for Spyking-circus. Density-based sorters (HerdingSpikes and IronClust), on the other hand, do not have a spike collision resolution strategy and this is reflected by their overall poorer performance. It is interesting to notice that Tridesclous, HDSort, YASS, and Kilsort 3, also using a template-matching-based procedure to resolve the spikes, are not properly resolving the temporal correlations even for dissimilar templates. Different template-matching strategies are probably the cause of the differences among sorters. For example, HDSort does not implement any strategy for spike collision resolution (Diggelmann et al., 2018), and that is reflected in the quick degradation of performance as template similarity increases. Kilosort uses a GPU-based implementation of the k-SVD algorithm (Aharon et al., 2006), used in matching learning as a dictionary learning algorithm for creating a dictionary for sparse representations. By doing so, it performs a reconstruction of the extracellular traces by optimizing both the templates and the spike times, which is an enhancement compared with what is done in Spyking-circus and Tridesclous. This might explain the boost in performance especially striking for templates with high similarity (similarity > 0.8).

### Generation of controlled spike collision simulated data

The results shown in the previous section have been obtained only in a particular regime of activity, with all neurons firing independently as Poisson sources with an average firing rate of 5 Hz. However, neurons usually do not fire independently of each other, but rather have intrinsic correlations, also depending on different brain areas, brain states, and species. In addition, the average firing rates can also largely vary depending on brain areas. As an example, it is well known that Purkinje cells in the cerebellum have a very high firing rate (Sedaghat-Nejad et al., 2021), that networks tends to synchronize their activity either in slow waves during sleep (Steriade, 2004), or during pathologic activity [such as epileptic seizures (Truccolo et al., 2011)]. Therefore, assessing how performances may vary during different conditions is important to generalize our observations.

In order to study how spike sorting is affected by correlations and firing rates, we used a mixture procedure (Brette, 2009) that allowed us to control precisely the shape of the auto-correlograms and cross-correlograms for the injected spike trains. More precisely, we decided to explore in a systematic manner three rate levels (5, 10, and 15 Hz), and three correlation levels (0%, 10%, and 20%). Note that the 5 Hz firing rate with 0% correlation corresponds to the scenario displayed in Figures 2 and 3.

**Figure 4.**
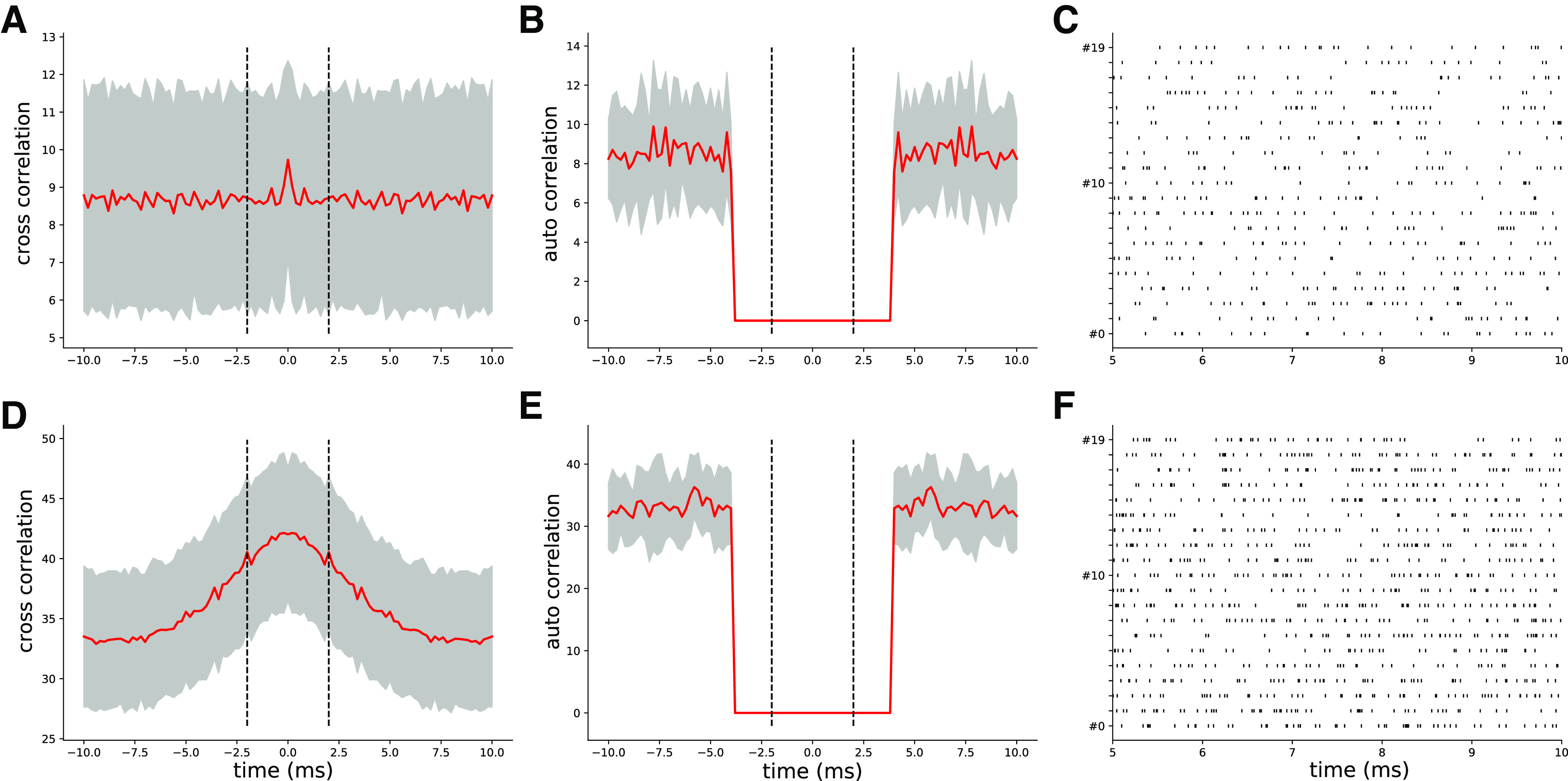
Controlling spike trains correlations and firing rates. ***A***, Average cross-correlograms between all pairs of distinct neurons firing as independent Poisson sources at 5 Hz (red curve, gray area represents the SD). ***B***, Same as ***A***, but for auto-correlograms. ***C***, Raster plot showing the activity of the uncorrelated neurons firing at 5 Hz. ***D–E***, Same as ***A–B***, but for a rate of 15 Hz and 20% correlation. ***F***, Raster plot showing the activity at 20% correlation and 15 Hz rate.

Figure 4 shows the average of cross-correlograms and auto-correlograms and the spike trains of a recording where cells are firing as independent Poisson sources at 5 Hz in panels *A–C* (and thus with 0% correlation, as shown by the flat average cross-correlograms in Fig. 4*A*) and at 15 Hz with 20% correlation (Fig. 4*D–F*). Although experimental recordings would contain a broader spectrum of firing rates and correlations, here we focus on assessing how different firing regimes affect spike sorting performance in a controlled setting. By varying these conditions, we wanted to challenge the internal clustering step of the spike sorting algorithms and see how generalizable are the results we observed in the previous section. One would expect that the increased density of spikes (both in terms of firing rates and of synchrony) should degrade the performance of the spike sorters by affecting both the clustering step and the template-matching step, which in turn would degrade the resolution of spike collisions. It is worthwhile noting that all the rates and correlation levels are homogeneous among neurons and only the templates are different.

### Do correlations and firing rates affect spike sorting of spike collisions?

To assess whether firing rate and spike train correlation affect spike sorting performance, we selected all unit pairs with a similarity >0.5. We first averaged the recall curves for all template similarities (i.e., we averaged the curves with similarity >0.5 shown in Fig. 3).

**Figure 5.**
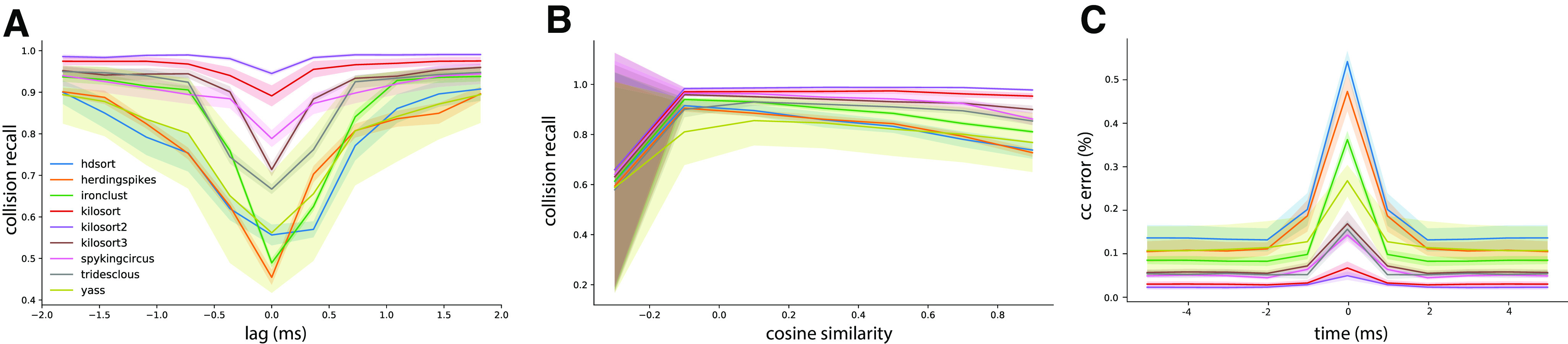
Spike sorting performance for different conditions. ***A***, Average collision recall over the nine conditions shown in Figure 6 (3 firing rate levels and 3 correlation levels) as function of the lag between spikes, for pairs of cells with cosine similarity higher than 0.5. The shaded area shows the SD over the conditions. ***B***, Similarly as ***A***, the average collision recall as function of the cosine similarity between pairs of cells. ***C***, Mean relative error between the ground-truth cross-correlograms and the estimated ones, for all sorters, averaged over all pairs with a similarity higher than 0.5.

**Figure 6.**
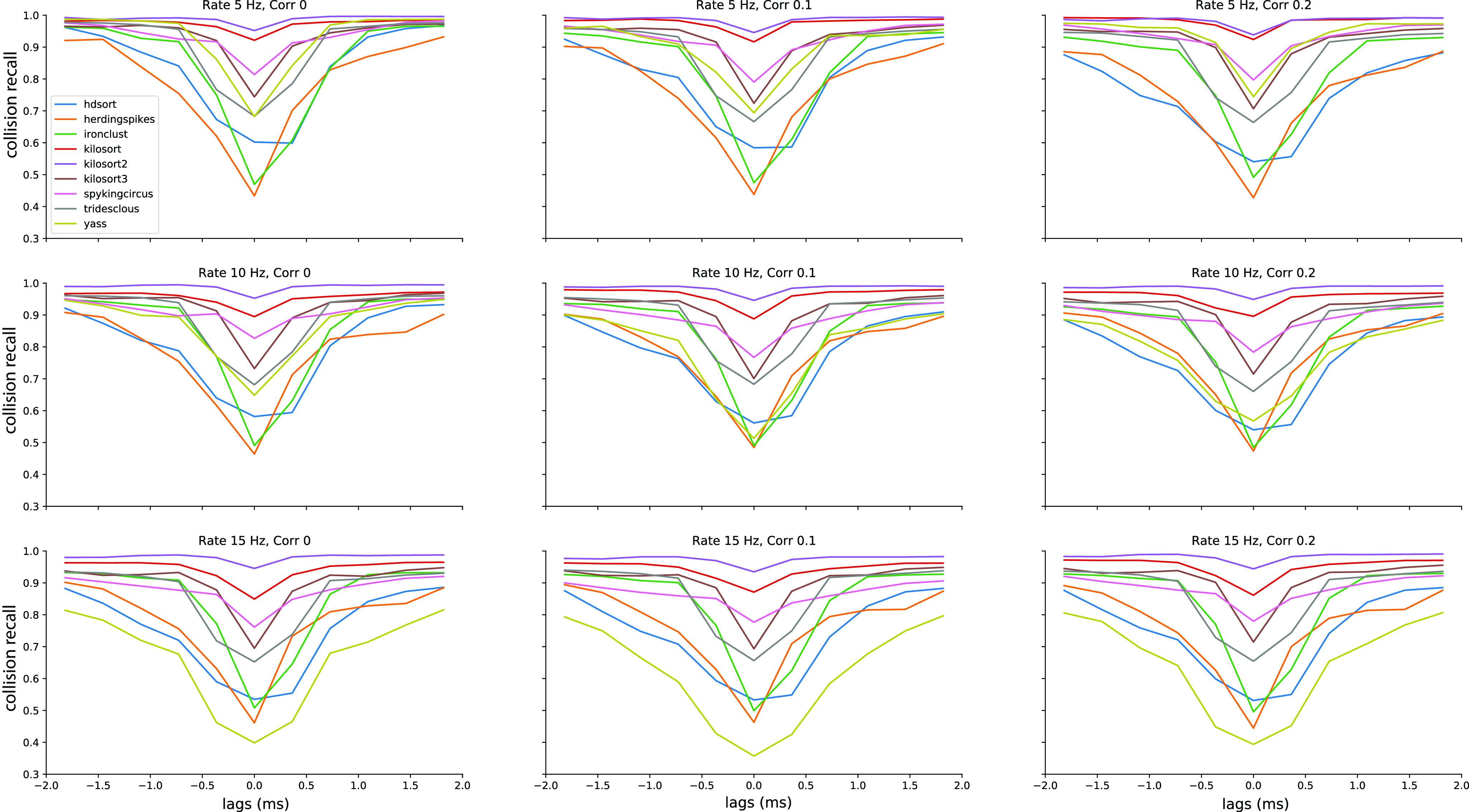
Average performances of the spike sorters as function of the temporal lags. Each panel shows the average collision recall for template pairs with a similarity above 0.5 for a different condition, in terms of firing rate and correlation levels.

In Figure 5*A*, we show the recall with respect to the spike lags averaged over all nine configurations (three firing rates x three correlations) for each sorter. The thick line represents the mean recall and the shaded area is the SD over different rate-correlation configuration. All sorters, except YASS, appear to have a very consistent curve (low SD) over different configurations and do not seem affected by changes in average firing rates and correlations in the spike trains. YASS’ large SD can be explained by looking at individual recall curves at different rate-correlation regimes (Fig. 6, yellow lines): the spike sorting performance degrades with increasing firing rates, but it does not seem to be strongly affected by increased correlation rates. However, we should stress that since the collision recall is a relative measure, the same value for a larger number of spikes (when firing rate is increased) means that overall, there are more misses for all sorters.

**Figure 7.**
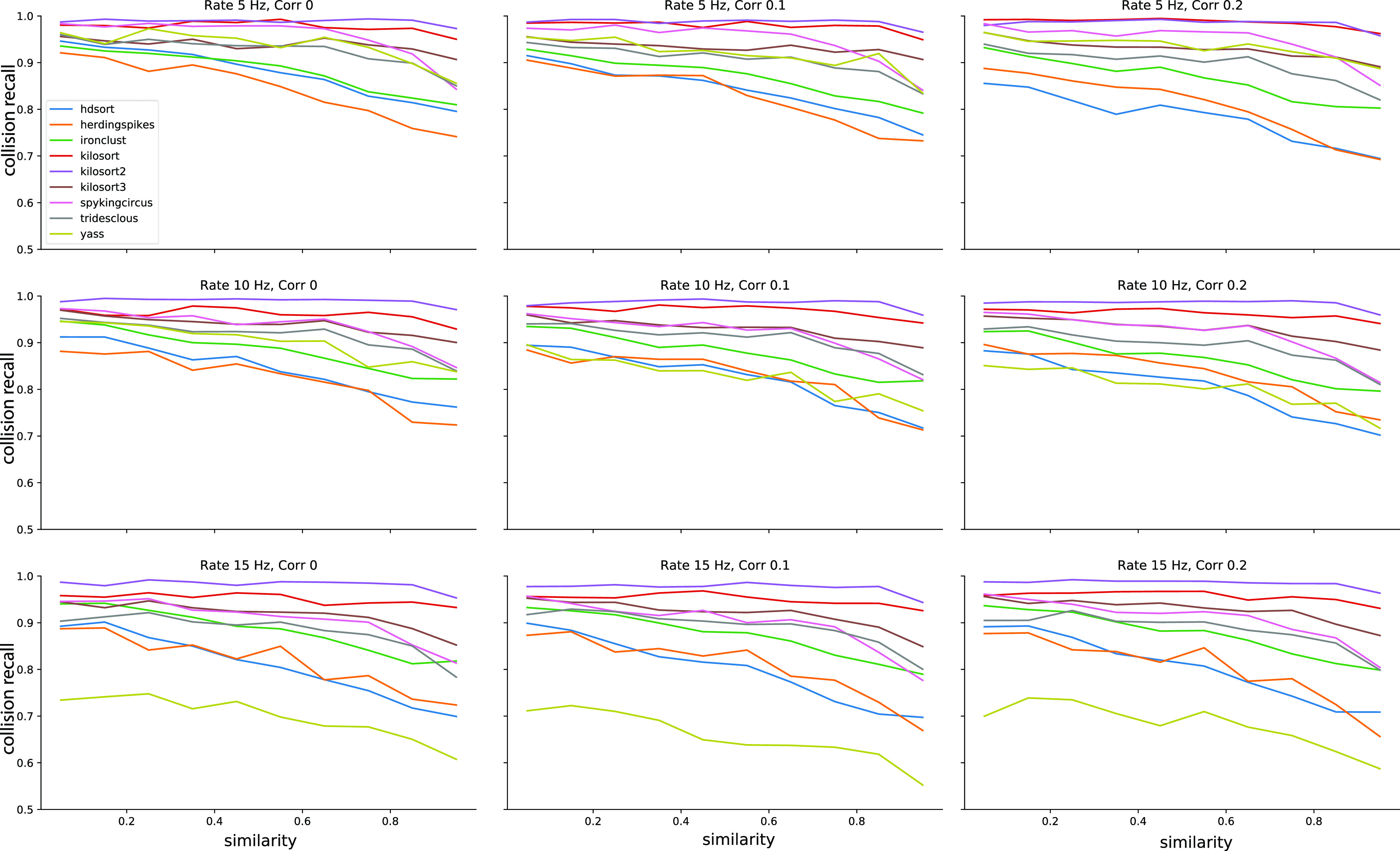
Average performances of the spike sorters as function of the template similarity. Each panel shows the average collision recall over all lags in [–2, 2] ms for a different condition, in terms of firing rate and correlation levels.

Similar considerations can be done by looking at the average recall with respect to template similarity (Fig. 5*B*). To construct these plots, we integrated the curves in Figure 3 over lags for different cosine similarities. Also in this case, the curves appear consistent (low SD) with the exception of YASS, for which recall is reduced with increased firing rate regimes (Fig. 7, yellow lines). It is worth noticing that when the cosine similarity becomes negative, all the sorters perform very poorly in properly resolving the overlaps. This could be explained by the fact that when a pair of templates is anti-parallel (Fig. 1*A*, left panel), a subset of electrodes might show a negative signal for one template and a positive signal from the other (because of return currents in the dendritic signals; Gold et al., 2009). Effectively, when a spike collision between the two spikes occurs, this would lower the amplitude of the negative peak, which could reduce the detectability of the spike.

The collision recall metric is mostly useful to obtain a quantitative insight on the behavior of the spike sorting algorithms, but how do these errors transpose in practical situations? To assess this, we measure the relative error (in percentage) between the ground-truth cross-correlograms and the ones computed from the spike sorting outputs. We then averaged these error curves among all recordings and experimental conditions (firing rates and synchrony levels). As shown in Figure 5, the error in the estimated cross-correlogram can be as large as >50% for small lags, and for some spike sorting algorithms such as HDSort, HerdingSpikes, or IronClust. Moreover, it is also worth noticing the baseline error rate is not the uniform across sorters. From this metric, we can again conclude that template-matching-based spike sorting algorithms such as Kilosort (1, 2, and 3), Spyking-circus, or Tridesclous are much better to resolve fine temporal correlations among neurons.

## Discussion

In this study, we showed in a systematic and quantitative manner how spatiotemporal correlations can be underestimated during spike sorting. Using synthetic datasets, we compared a large diversity of modern spike sorters and showed how they behaved as function of the similarity between the templates and the temporal lags between spikes. As expected, the closer the spikes are in time, the harder is it, for all sorter, to properly resolve the overlaps. However, more interestingly, the more similar the templates are, the higher the failures are. These failures are striking especially for spike sorters that are not relying on template-matching-based approaches (HerdingSpikes, IronClust). For the ones using a template-matching-based approach (Kilosort, Spyking-circus, Tridesclous, HDSort), the problem is less pronounced (with the exception of HDSort) but still present, and therefore this phenomenon should be taken into account when making claims about the synchrony.

To our surprise, the global behavior of the spike sorters did not depend much on the overall firing rate and/or the correlation levels. This allows us to generalize the findings and we think that the quantitative results shown here could be translated to various *in vitro* or *in vivo* recordings from different brain regions and species. As shown in Figure 5, while the variability over different conditions is rather high for some algorithms, template-matching-based algorithms tend to be rather robust and overall better in resolving spike collisions. This is a very encouraging sign toward a unified and reproducible automated solution for spike sorting (Buccino et al., 2020; Magland et al., 2020), agnostic of the recording conditions.

The results shown in the paper were obtained with purely artificial recordings, since we need exhaustive information on the ground-truth spiking activity of all neurons to quantitatively compare and benchmark different spike sorters. However, it would be interesting to generalize these observations with real recordings, assuming one would have a proper ground truth at the population level. Indeed, such a ground truth is needed to compute the collision recall and see how sorters behave as function of lags and similarities between templates. To our knowledge, such a ground truth does not exists (Neto et al., 2016; Diggelmann et al., 2018; Yger et al., 2018). While one could try to generate an “approximated” ground truth by combining the output of several spike sorters with an ensemble spike sorting approach (as in Buccino et al., 2020), the disagreements among sorters are currently so high that this process is hard if not impossible, if one want to sample from a large number of pairs.

While missing spikes for very dissimilar templates and small lags is problematic, the errors made for very similar templates may be less frequent depending on the probe layout and neuronal preparation. Indeed, such errors strongly depends on the distribution of template similarities between all pairs of recorded cells, and this distribution might differ from recording to recording. For example, in the retina (Wässle, 2004) one would expect highly synchronous cells, of the same functional type, to be far apart from each other because of an intrinsic tiling of the visual space. Such properties are unknown *in vivo* or in cortical structures, but might bias the distribution of template similarities between nearby neurons, and thus modify the estimation of collision recalls.

## Synthesis

Reviewing Editor: Arvind Kumar, KTH Royal Institute of Technology Decisions are customarily a result of the Reviewing Editor and the peer reviewers coming together and discussing their recommendations until a consensus is reached. When revisions are invited, a fact-based synthesis statement explaining their decision and outlining what is needed to prepare a revision will be listed below. The following reviewer(s) agreed to reveal their identity: Marius Pachitariu, Jason Chung.

Synthesis

This study provides a systematic and thorough evaluation of template matching in resolving overlapping spikes. Overall both the reviewers have found that the manuscript will be a valuable addition to the literature on spike sorting. But they have raised some points that need to be addressed.

It is unclear why different template matching algorithms perform so differently in terms of spike overlap. Reviewers do not agree with the authors’ reasoning behind the difference [see below in the reviewers’ comments]. To address this the reviewers’ suggest that you try to separate the clustering from the template matching completely, i.e. give the algorithms the ground truth templates and see how well they do then

Reviewers also noted the high-density probes definitely do not “dramatically increase the amount of overlapping spikes”. They just enable dense sampling of such overlapping spikes. But perhaps more importantly, by recording enough channels with independent sampling of the waveforms high-density problem in fact allow us to actually solve the spike overlap problem,

Several other questions have also remained unaddressed e.g.

How does the collision recall/error rates vary as a function of distance?

How many spikes are necessary to appropriately separate two colliding units?

How long are the simulated recordings?

Addressing these questions may help in improving the interpretability of the results. In this regard the distance between template peaks may also help aid interpretability for other electrode array configurations

So we would like to invite you to revise the manuscript to address the aforementioned concerns.

To aid the revision, the comments from the two reviewers are appended below.

Reviewer #1

In this study, the authors compare the ability of different spike sorting algorithms to resolve coincidental spikes between neurons very close to each other in tissue. This study confirms that template matching methods generally perform much better in this regard compared to other approaches. Indeed template matching methods were developed to address this specific issue, so it should be no surprise that they do address it. Nonetheless, the thorough analysis across spike sorters is valuable to the field and a good confirmation that some algorithms work the way they were supposed to. I think more could be done to elucidate some remaining mysteries (see below) and to turn these analyses into a set of actionable recommendations for future spike sorting algorithms.

I think the main mystery is why different template matching algorithms perform so differently in terms of spike overlap. The algorithms these methods all use are thought to be fairly similar. An argument is given in the manuscript that Kilosort 1 and 2 perform better because they use orthogonal instead of greedy matching pursuit, but this is not true. They actually both use greedy matching pursuit as well. In addition, Kilosort 1/2/3 uses the exact same algorithm for template matching, with minor differences in default parameters. Different versions of Kilosort only differ in the preprocessing part (i.e. drift correction) and in the clustering part (i.e. scaled k-means for KS 1/2 and bimodality pursuit for KS 3).

I think it’s important to figure out where the differences come from, because it questions the premise of these analyses, i.e. that we’re looking at spike collision performance irrespective of the accuracy/precision/recall metrics. Instead, I think the clustering step itself makes a big difference, and I recommend you try to separate the clustering from the template matching completely, i.e. give the algorithms the ground truth templates and see how well they do then. If there are still major differences between methods (especially between KS1/2/3), that would imply that relatively small details matter, in the different implementations of template matching and/or parameters.

Finally, one important comment about the motivation used in several places: high-density probes definitely do not “dramatically increase the amount of overlapping spikes”. This is physically not possible, the spike overlap is due to the overlap of the electrical fields, which is a fixed property of the neurons. High-density probes do the opposite, they allow us to actually solve the spike overlap problem, by recording enough channels with independent sampling of the waveforms.

Marius Pachitariu

Reviewer #2

The work focuses on evaluating modern spike sorters specifically with respect to the issue of spike collision - simultaneous spikes from two neurons detected on the same electrode array. The authors use a synthetic ground-truth dataset (generated by MEArec) where true error rates can be quantified. They then use the spikeinterface to compare among 9 different spike sorters. From this, they are able to conclude that in general, template-based methodologies and sorters designed with specific measures to counteract collision perform better than others. Interestingly, they also find that the rate and correlations among colliding units do not seem to influence the results. Overall, I commend the authors and support this work on an increasingly relevant topic within spike sorting and single-unit electrophysiology. There are a few areas where additional analyses should be completed which would improve the practical relevance to electrophysiologists.

Major

1)Spatial distance and collision

The authors use two methods to quantify the similarity of the templates, the cosine similarity as well as the time lag. The distance between template peaks would help aid interpretability for other electrode array configurations where electrode size/density may differ from the synthetic data here. With higher density arrays one would expect to have better spatial separation between templates (and thus lower cosine similarity), but this complex interaction is not easily interpreted from what is currently shown. What does collision recall/error rates look like as a function of distance?

2)Number of spikes

How many spikes are necessary to appropriately separate two colliding units? How long are the simulated recordings? The spike train correlations (Figure 5 supplement 1) get at this by showing the rates and correlations, but the number of spikes in the colliding clusters would aid interpretability for lower rate units.

Minor

3)Example templates for similarities 0.4-0.8

Figure 5 supplement 2 shows decline in performance as cosine similarity rises. Currently, figure 1B shows 3 example templates of similarities -0.2, 0, and .086. It would be useful to see more examples spanning the range where performance declines, 0.4 to 0.8.

4)Practical solutions/guidance for identifying collision related errors

Is there a way that physiologists can identify potentially problematic collisions in their datasets?

## Author Response

Response to the reviewers: How do spike collisions affect spike sorting performance?

Dear Editor and Reviewers,

We would first like to thank the reviewers for their valuable feedback in reviewing our review titled "How do spike collisions affect spike sorting performance?”.

In the following we will address each referee’s comments point by point, using different fonts and colors to clearly show the implemented changes: previous manuscript, the new manuscript, the reviewers’ comments, and our response.

Reviewer 1

Major comments

(...) I think it’s important to figure out where the differences come from, because it questions the premise of these analyses, i.e. that we’re looking at spike collision performance irrespective of the accuracy/precision/recall metrics. Instead, I think the clustering step itself makes a big difference, and I recommend you try to separate the clustering from the template matching completely, i.e. give the algorithms the ground truth templates and see how well they do then. If there are still major differences between methods (especially between KS1/2/3), that would imply that relatively small details matter, in the different implementations of template matching and/or parameters.

We thank the reviewer for this comment. We agree on the fact that a proper separation of the clustering and the template-matching steps would be ideal to properly benchmark the performance against spike collisions, since these are mainly resolved at the template-matching step. However, in the current state of spike sorting tools, breaking down these two steps for each sorter would require us to have a proper knowledge of the internal machinery of all the sorters, in order to be able to properly separate the two steps and plug in the “final” templates, bypassing the clustering, before any further template-matching procedure. We would need to re-implement all the steps previous to template-matching, making sure to keep into consideration the exact fine details that allows each template-matching based sorter to produce the final catalogue of templates(e.g., filtering, whitening, dimensionality reduction, sparsity, etc.) in order to be sure not to artificially decrease the performance. Moreover, for some sorters, the templates are not simply extracted as mean or median over a sample of snippets, and might results from an optimization procedure, either intrinsically mixed with the deconvolution procedure [3], or relying on deep networks that can not be easily examined [2]. Nevertheless, in the paper, we tried to partially address this dichotomy by showing that all ground-truth templates have been correctly discovered by most of the algorithms: in Figure 2C we compare the average accuracy of the sorters over several recordings to the average cosine similarity values between the real ground-truth templates and the ones found by the sorters. As it can be seen in this plot, the fact that for most of the template-matching based sorters, the cosine similarity is very high means that the sorters are finding the correct templates, and that, in turn, the core of the problem seems to lie in the template-matching procedure. The Figure has been updated to provide a zoomed in panel of the well-discovered templates and we added the following sentence in the relevant portion of the text: “The average cosine similarity between discovered and ground-truth templates is larger than 0.97 for most of the template-matching based sorters (Spyking-circus, Kilosort 1/2/3, IronClust, Tridesclous), so we can safely assume that most of the errors are not due to the clustering step”. Of course, one cannot rule out that subtle differences in the templates obtained after the clustering could be amplifying the errors, but we strongly believe that establishing a clear separation is out of the scope of this paper.

In fact, we would like to point to the reviewer that we are already working, within the SpikeInterface initiative [1], towards the suggested direction. The goal is to propose a complete and modular spike sorting pipeline such that algorithmic steps such as clustering and deconvolution/template-matching can be easily isolated and benchmarked. In such a framework, we are already able to compare various engines for template-matching, and we hope to be able to provide soon, in follow-up studies, more insights on the issue raised by the reviewer.

An argument is given in the manuscript that Kilosort 1 and 2 perform better because they use orthogonal instead of greedy matching pursuit, but this is not true. They actually both use greedy matching pursuit as well. In addition, Kilosort 1/2/3 uses the exact same algorithm for template matching, with minor differences in default parameters. Different versions of Kilosort only differ in the preprocessing part (i.e. drift correction) and in the clustering part (i.e. scaled k-means for KS 1/2 and bimodality pursuit for KS 3)

We apologize for the unclarity in the original manuscript and we would like to highlight that this is not what we wanted to convey in the text. In the manuscript, we compared the performance of Spyking-circus (using a matching pursuit approach), to the one of Kilosort, using an orthogonal matching pursuit approach. Because both methods are greedy, we removed such a terminology in the paper. What we meant is that the difference between the two is that in the latter, each time a new template is added to the reconstruction of the signal, all the amplitudes are updated, while this is not the case for Spyking-circus and Tridesclous. Therefore, one could expect Kilosort (1/2/3) to perform well even for non-orthogonal templates (thus with high cosine similarity). This is somehow what is shown in Figure 3 for Kilosort 1 and 2, but surprisingly not for Kilosort 3. The reason why this is the case requires more investigation. As suggested by the reviewer, it could be either because of some subtle differences in the default parameters of these algorithms, and/or because of the different clustering step. The only certainty is that all Kilosort versions are finding templates very similar to the ground-truth ones, as one can see from Figure 2C.

Finally, one important comment about the motivation used in several places: high-density probes definitely do not “dramatically increase the amount of overlapping spikes”. This is physically not possible, the spike overlap is due to the overlap of the electrical fields, which is a fixed property of the neurons. High-density probes do the opposite, they allow us to actually solve the spike overlap problem, by recording enough channels with independent sampling of the waveforms.

We thank the reviewer for this comment. We agree with the reviewer that high-density probes do not increase the amount of overlapping spikes. What we meant was that with the increased density of the recording channels, the probability of isolating neurons via spike sorting is also higher, and so is, as a direct consequence, the probability of having to deal with overlapping spikes (either in space or in time). But for the sake of the clarity, this has been removed in the manuscript, and the abstract has been modified to remove this statement. A B C D HD HS IC KS KS2 KS3 SC TDC YS HD HS IC KS KS2 KS3 SC TDC YS 0.98 1.0 0.9 Figure R1: Spike sorting performance. A) Average run times over 5 different recordings (see Methods) for all the tested sorters. Errors bars indicate the standard deviation over multiple recordings.

B) Average number of cells found by the sorters that are either well detected, redundant, overmerged or considered as false positive (see Methods). Error bars indicates standard deviation over multiple recordings. C) The average cosine similarity between templates found by the sorters and ground-truth templates, as function of the accuracy for the given neurons. Ellipses shows standard error of the means in cosine similarity (x-axis) and accuracy (y-axis). D) Average metrics (accuracy, precision, recall, see Methods) for all the sorters. Error bars show standard deviation over multiple recordings.

Reviewer 2

Major comments

The authors use two methods to quantify the similarity of the templates, the cosine similarity as well as the time lag. The distance between template peaks would help aid interpretability for other electrode array configurations where electrode size/density may differ from the synthetic data here. With higher density arrays one would expect to have better spatial separation between templates (and thus lower cosine similarity), but this complex interaction is not easily interpreted from what is currently shown.

What does collision recall/error rates look like as a function of distance?

We thank the reviewer for the comment. We would first like to clarify that the time lag is not used to quantify the similarity between templates, but only as a measure of how closely the spikes from two neurons overlap in time. Instead, we used solely the cosine similarity as a measure of how “similar” templates are, since it appeared to us to be a relevant quantity to probe the spike sorting errors with respect to spike collisions.

As one can see in Figure 1D, there is, at least in our synthetically generated recordings, a clear relationship between the physical distance between cells (either estimated or computed from the in silico ground-truth cell locations) and the cosine similarities of their templates. The closer two cells are, the higher the cosine similarity is, simply because the same channels are often co-activated. We agree with the reviewer that cosine similarity would also suffer form the curse of dimensionality, i,e., with very high density probes the range of values would probably be smaller. Nevertheless, it would still allow one to rank templates based on their similarity. Instead, using the physical distance between the electrodes recording the largest peak (as the reviewer suggests) as a similarity measure could be misleading. For example, if two neurons are close to each other their physical distance could be very small (for example if their peak is on the same electrode). However, their templates could be very dissimilar because of morphological and electrical differences and, in our view, the similarity measure should be able to account for these differences (and cosine similarity does).

How many spikes are necessary to appropriately separate two colliding units? How long are the simulated recordings? The spike train correlations (Figure 5 supplement 1) get at this by showing the rates and correlations, but the number of spikes in the colliding clusters would aid interpretability for lower rate units.

The simulated recordings are 30 minutes long, and this has been added in the Simulated Dataset section of the Methods. We believe that a duration of 30 minutes is long enough for all sorters to be able to correctly find individual templates of the neurons in the recording, as confirmed by the performance metrics in Figure 2 (in particular panel C). Provided that the sorters found the correct templates for the ground-truth neurons, the errors are shown as percentage of the colliding spikes and the absolute number ofcolliding spikes should not affect the error rate. For template-matching based sorters, in principle, if neuron A and B have been discovered by the clustering and their templates have been correctly estimated, the template-matching phase should be able to resolve even a single collision. This is not the case for density-based sorters, whose performance is in fact poorer with respect to template-matching sorters. To respond to the reviewer’squestion, the absolute number of colliding spikes for every pair of neurons can be estimated from Figure 4, which shows the cross-correlograms between neurons. The number of colliding spikes for any pair (note that the spiking activity and correlations are homogeneous for each recording) is the integral over [−2ms, 2ms] (x-axis) of the number of collisions (y-axis), so it is ∼90 for (5 Hz - no correlation, Figure 4A) and ∼400 for (15 Hz - 0.2 correlation, Figure 4D). However, we do not believe that these values are relevant for the results since they do not impact the collision recall measures, which are expressed in percentage.

Minor comments

Figure 5 supplement 2 shows decline in performance as cosine similarity rises. Currently, figure 1B shows 3 example templates of similarities -0.2, 0, and .086. It would be useful to see more examples spanning the range where performance declines, 0.4 to 0.8.

Since Figure 1 is intended to be an illustration of the behavior of the cosine similarity, we decided to only show three pairs, with representative values. While we do agree with the reviewer that every pair is different, we feel that adding too many pairs in the range[0.4,0.8] might overload the figure. However, we agree that adding one more pair with intermediate cosine (≃ 0.5) could be informative, so we modified the first figure of our main paper accordingly. In addition, in Figure R2 we display additional random examples of pairs of templates with cosine similarities in the [0.4, 0.8] range.

Is there a way that physiologists can identify potentially problematic collisions in their datasets?

We believe that this is a really complicated question that would deserve additional studies to be properly addressed. In this paper, we wanted to benchmark the performance of spike sorting algorithms specifically against spike collisions. In fact, although novel sorters claim to be able to handle spike overlapping spikes, this was never tested in practice. In order to do so, we used artificially generated ground-truth recordings, 20 10 0 10 20 100 50 0 50 100 150 (#19, #11) similarity 0.788973 20 10 0 10 20 100 50 0 50 100 150 (#19, #13) similarity 0.547705 20 10 0 10 20 150 100 50 0 50 100 150 (#14, #2) similarity 0.541952 20 10 0 10 20 100 50 0 50 100 150 (#13, #17) similarity 0.54257 20 10 0 10 20 100 50 0 50 100 150 (#19, #10) similarity 0.59202 20 10 0 10 20 100 50 0 50 100 150 (#5, #9) similarity 0.791252 20 10 0 10 20 150 100 50 0 50 100 150 (#8, #19) similarity 0.528936 20 10 0 10 20 150 100 50 0 50 100 150 (#2, #4) similarity 0.609138 20 10 0 10 20 150 100 50 0 50 100 150 (#15, #2) similarity 0.675592 Figure R2: Example of templates randomly selected with cosine similarities between [0.4, 0.8]. that allowed us to perform such exhaustive benchmarking effort knowing the underlying ground-truth activity. While the template-matching algorithms are clearly better, they still make some errors, especially for very close/synchronous cells, with high cosine similarity between their templates. We do not believe that one could identify potentially problematic collision events in experimental recordings if they are missed by the spike sorter. However, we hope that this work will push spike sorting developers to improve existing tools and not to consider the spike collision problem as solved. In addition, we think that physiologist should be aware that spike collisions can be problematic to resolve, especially in research that makes claims on very fine synchronous patterns at the population levels.

References

[1] A. P. Buccino, C. L. Hurwitz, S. Garcia, J. Magland, J. H. Siegle, R. Hurwitz, and M. H. Hennig. Spikeinterface, a unified framework for spike sorting. Elife, 9:e61834, 2020.

[2] J. Lee, C. Mitelut, H. Shokri, I. Kinsella, N. Dethe, S. Wu, K. Li, E. B. Reyes, D. Turcu, E. Batty, et al. Yass: Yet another spike sorter applied to large-scale multi-electrode array recordings in primate retina. bioRxiv, 2020.

[3] M. Pachitariu, N. A. Steinmetz, S. N. Kadir, et al. Fast and accurate spike sorting of high-channel count probes with kilosort. In Advances in Neural Information Processing Systems, pages 4448- 4456, 2016.
